# Genetics and Pathophysiology of Neurodegeneration with Brain Iron Accumulation (NBIA)

**DOI:** 10.2174/157015913804999469

**Published:** 2013-01

**Authors:** Susanne A Schneider, Petr Dusek, John Hardy, Ana Westenberger, Joseph Jankovic, Kailash P Bhatia

**Affiliations:** 1 Department of Neurology; University of Kiel, 24105 Kiel, Germany; 2 Sobell Department of Motor Neuroscience and Movement Disorders, Institute of Neurology, UCL, Queen Square, London WC1N 3BG, UK; 3 Department of Neurology and Center of Clinical Neuroscience, Charles University in Prague, 1st Faculty of Medicine and General University Hospital, Prague, Czech Republic; 4 Department of Molecular Neuroscience, Institute of Neurology, UCL, Queen Square, London WC1N 3BG, England; 5 Schilling Section of Clinical and Molecular Neurogenetics at the Department of Neurology, University of Lübeck, Lübeck, Germany; 6 Parkinson's Disease Center and Movement Disorders Clinic, Department of Neurology, Baylor College of Medicine, Houston, TX 77030, USA

**Keywords:** Ceramide, dystonia, iron, NBIA, parkinsonism, MPAN, PKAN, PLA2G6.

## Abstract

Our understanding of the syndromes of Neurodegeneration with Brain Iron Accumulation (NBIA) continues to grow considerably. In addition to the core syndromes of pantothenate kinase-associated neurodegeneration (PKAN, NBIA1) and PLA2G6-associated neurodegeneration (PLAN, NBIA2), several other genetic causes have been identified (including FA2H, C19orf12, ATP13A2, CP and FTL). In parallel, the clinical and pathological spectrum has broadened and new age-dependent presentations are being described. There is also growing recognition of overlap between the different NBIA disorders and other diseases including spastic paraplegias, leukodystrophies and neuronal ceroid lipofuscinosis which makes a diagnosis solely based on clinical findings challenging. Autopsy examination of genetically-confirmed cases demonstrates Lewy bodies, neurofibrillary tangles, and other hallmarks of apparently distinct neurodegenerative disorders such as Parkinson’s disease (PD) and Alzheimer’s disease. Until we disentangle the various NBIA genes and their related pathways and move towards pathogenesis-targeted therapies, the treatment remains symptomatic.

Our aim here is to provide an overview of historical developments of research into iron metabolism and its relevance in neurodegenerative disorders. We then focus on clinical features and investigational findings in NBIA and summarize therapeutic results reviewing reports of iron chelation therapy and deep brain stimulation. We also discuss genetic and molecular underpinnings of the NBIA syndromes.

## INTRODUCTION

Delicate regulation of iron metabolism is important for maintaining good health as iron deficiency or iron overload can lead to disease [1]. Abnormalities in brain iron metabolism with excess iron levels give rise to a variety of neurodegenerative diseases. In recent years, advances in neurogenetics, such as powerful autozygosity mapping, have led to identification of several genes that are associated with disturbed brain iron metabolism and may cause the syndromes of Neurodegeneration with Brain Iron Accumulation (NBIA). These present clinically with a progressive hypo- and/or hyperkinetic movement disorder and pathologically with excessive iron deposition in the brain, particularly affecting the basal ganglia, mainly the globus pallidus (GP). The two core syndromes, accounting for most cases, are the neuroaxonal dystrophies pantothenate kinase-associated neurodegeneration (PKAN, formerly known as Hallervorden-Spatz disease), currently classified as NBIA type 1 and PLA2G6-associated neurodegeneration (PLAN), classified as NBIA type 2. However, further causative genes underlying other, much scarcer, NBIA syndromes have been identified recently (Table **1**) [2,3]. 

This rapid development in this field prompted us to present this review. Our aim is to provide an overview of physiological metabolism of brain iron and recapitulate the historic milestones in research of the brain iron metabolism, but then focus on the clinical features, investigational findings and therapeutic results, as well as genetic and molecular underpinnings of the syndromes of disturbed brain iron metabolism. We wish to particularly highlight the broad and expanding clinical spectrum of these complex disorders. We conclude by emphasizing that diagnosis based purely on clinical grounds is challenging because phenotypes of one distinct disorder are broad, partly age-dependent, and ranging from mild to severe. Moreover, there is often phenotypic overlap of the different NBIA disorders as well as with other diseases. This may be a consequence of fairly uniform pattern of degeneration in NBIA disorders due to the propensity of several brain structures for accumulation of toxic iron levels. 

## NORMAL BRAIN IRON METABOLISM 

Iron is essential for many brain functions, including energy production, DNA synthesis and repair, phospholipid metabolism, myelination and neurotransmitter synthesis [1,2,4]. 

Between different cell types and between different brain regions iron content varies considerably with high concentrations in oligodendrocytes, particularly in the cortex and cortico-subcortical junction, and low concentrations in neurons and astrocytes [5]. The iron levels vary in microglia, since these cells store and release iron according to tissue metabolic needs [6]. Brain iron levels are also age-dependent. Essentially no iron is detectable in the brain at birth [7-9], but iron then accumulates during development. Notably, iron accumulation also occurs within areas of myelination [10]. In adulthood and old age, slow gradual increase of iron deposition is observed mostly in microglia and astrocytes, [5] and this is particularly prominent in the GP, red nucleus, substantia nigra (SN) pars reticulata, dentate nucleus, and putamen but to a lesser degree also in the caudate, thalamus and frontal gray matter as documented by histochemistry [11-13] and magnetic resonance imaging (MRI) studies [14-16]. As early as in the 1920s first systematic studies of iron in the human brain demonstrated such differences in concentration with highest levels in the GP and SN pars reticulata [7]. Why iron selectively accumulates with increasing age, remains poorly understood. It has been hypothesised that dysfunction of the blood brain barrier [17,18] may allow uncontrolled entry of iron to predisposed areas.

Another possibility is that iron accumulation is triggered by apoptotic cascade or results from cellular damage. It has been shown that ceramide-mediated apoptosis is dependent on increased cellular iron uptake [19]. It is thus conceivable that brain iron accumulation observed in neurodegenerative disorders may be an unspecific marker or epiphenomenon of apoptosis. In a rat model, iron accumulation and neuronal loss have been observed in the substantia nigra following lesioning of the striatum/GP [20]. Degeneration of nigral neurons in this study might have been caused by their denervation and subsequent loss of trophic support. There is experimental evidence that cortical pathology or white matter lesions leading to downstream deafferentation of the basal ganglia may eventually lead to iron accumulation in these structures. Yet, demyelinating or vascular white matter lesions may lead to iron accumulation through a different mechanism. Iron is crucial for myelination, but high amounts of iron released from damaged oligodendrocytes apparently cannot be used for remyelination. Instead, iron uptake from plasma may be necessary while the “old” unrecyclable iron is translocated downstream into basal ganglia [21]. Thus, damaged white-matter may be a source of abnormal iron accumulation and recent studies assessed the role of iron in multiple sclerosis and other disorders affecting white matter [22-24]. Apart from demyelination, age-related vascular changes and mitochondrial dysfunction leading to hypoxia may also enhance cellular iron uptake through activation of hypoxia-inducible-factor-1 (HIF1) [25,26]. Mitochondrial biogenesis, which occurs in response to hypoxia in neurons, is a compensatory mechanism maintaining energy production [27]. Increased iron uptake may thus be required for enzymes involved in energy production in mitochondria (see [28]). 

Overall, it remains unclear whether increased brain iron content, documented in many neurodegenerative diseases, is a direct cause of neurodegeneration, a secondary event in a pathophysiologic cascade, or just a nonspecific marker of neurodegeneration.

## MILESTONES IN RESEARCH OF IRON ACCUMULATION UNDERLYING DISEASE

Many sporadic and genetic disorders demonstrate increased brain iron load. The first explicit description dates back to the 1920s, when Hallervorden reported high levels of brain iron identified in a family affected by a progressive neurological disorder dominated by extrapyramidal features [29]. Histology, furthermore, revealed “spheroid bodies” - usually roundish, homogeneous or faintly granular bodies, measuring up to 100 mu in diameter [29]. Within a few years similar cases were reported and variants were recognized, including early-onset forms referred to as “infantile neuroaxonal dystrophy” (INAD) [28]. 

An important milestone in the evaluation of these diseases was the development of high-field MRI in the 1980s which allowed for non-invasive neuroimaging *in vivo. *Iron is depicted as hypointensity on T2-weighted images due to shortening effect on relaxation time [7,30] with a linear relation between T2 relaxation rate and tissue iron concentration in postmortem brains. Thus, MRI has become an important research and diagnostic tool for disorders of iron metabolism including the NBIAs. However, to a milder degree iron is also present in other conditions including Parkinson’s disease [31,32] (mainly affecting the substantia nigra) and atypical parkinsonian disorders, Friedreich ataxia and multiple sclerosis (for extensive review see [33]).

## NBIA TYPE 1 - PKAN 

The core syndrome among the NBIA disorders is PKAN, due to mutations in the *PANK2* gene located on chromosome 20p. The world-wide prevalence has been estimated at 1: 1.000.000. In some areas, such as the Dominican Republic, near the town of Cabral, PKAN appears more commonly (1:10:000) due to a founder effect. Of all NBIAs, PKAN accounts for approximately half, although there remains uncertainty of the diagnosis in cases reported prior to gene identification which were based on clinical or pathological findings. Identification of the gene revealed a broad clinical spectrum with an age-dependent phenotype: in the classic presentation the age at onset is early, whereas patients with a later onset often show atypical clinical features. 

### Classic Presentation of PKAN

In the classic variant, onset occurs before the age of 6 years in almost 90% [34,35] often with gait difficulty as the presenting symptom [35]. The phenotype is further characterized by pyramidal (spasticity, hyperreflexia, extensor plantar toe response) and extrapyramidal features with prominent dystonia, often with predominant orolingual-mandibular [36] involvement. PKAN is thus one of the main differential diagnoses to consider in patients with severe tongue protrusion dystonia [36]. Other extrapyramidal features such as parkinsonism, chorea and a variety of neuropsychiatric features including attention deficit hyperactivity disorder [37], cognitive decline, and behavioral changes [38], including regressive and pseudobulbar affect are seen [39]. Oculomotor abnormalities are common, partially caused by mid-brain degeneration [40]. Among those, saccadic pursuit as well as hypometric or slowed saccades in the vertical plane are frequent findings. Supranuclear gaze vertical palsy has also been reported in a gene-proven case – a sign also associated with Niemann Pick disease and Kufor Rakeb disease (see below) [41]. Square wave jerks and poor convergence may be present in some. In a systematic study of ten patients there was insufficient suppression of the vestibulo-ocular reflex in seven examined patients [40]. Furthermore, eight had sectoral iris paralysis and partial loss of the papillary ruff with similarities to Adie’s pupils in both eyes [40]. Interestingly, similar pupil abnormalities have been observed in other brain storage diseases [40]. Only four of the ten had a pigmentary retinopathy, but around 70% of patients had abnormal electroretinograms ranging from mild cone abnormalities to severe rod-cone dysfunction [40]. None had optic atrophy (which may be more characteristic of other NBIA subtypes, including the PLA2G6-variant, see below). PKAN takes a progressive course with affected children usually becoming wheelchair-bound within a few years. 

### Late-onset (Atypical) PKAN

Gene-proven cases with adult onset (in the 20s and 30s) have been reported [35,42,43]. However, it is speculated that many cases are probably not being recognized, in particular because the phenotype may be somewhat atypical. For example, unilateral dystonic tremor and focal arm dystonia have been reported as first sign. In others extrapyramidal features and retinopathy may be less prominent, but cognitive decline and psychiatric features may be the leading symptoms [35,43-45]. Overall, compared to the classical form, motor involvement tends to be less severe. 

### Investigations in PKAN

Sleep analysis in PKAN revealed a reduction of total sleep time [46]. In contrast with other neurodegenerative diseases, however, REM sleep abnormalities, especially REM sleep behavior disorder, as well as significant apnea/hypopnea were absent. 

**Fig. (1):**
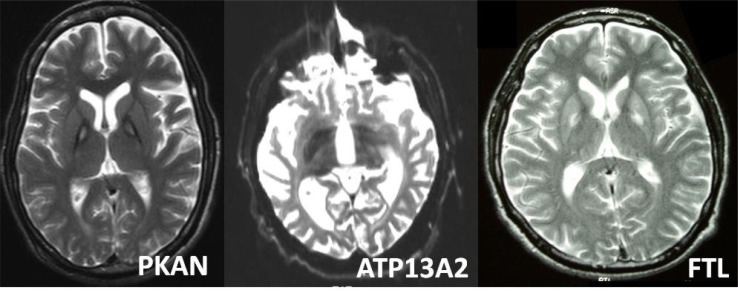
Examples of brain MR imaging in NBIA disorders; showing a case of pantothenate kinase associated neurodegeneration (PKAN) (left), Kufor Rakeb disease (due to ATP13A2 mutations) (center) and neuroferritinopathy (due to FTL mutations) (right). In PKAN there is a classic eye of the tiger sign. Iron accumulation affects the putamen and caudate in our Kufor Rakeb disease patient. In this gene-proven neuroferritinopathy patient there is iron deposition in the basal ganglia, with a slight hint of thalamic involvement. Reproduced from [3].

The importance of high-field MRI, [7,30] using particularly the iron sensitive T2*-weighted MR images, lies in the possibility to pick up a characteristic imaging pattern, corresponding to the iron accumulation in the anterior-medial part of the globus pallidus, in some extending into the knee of the internal capsule [47,48]. The presence of a central hyperintensity within a surrounding area of hypointensity in PKAN led to the description as “eye of the tiger sign” (Fig. **1**). Additional subthalamic and nigral iron has also been observed [47,48]. Importantly, the development of the MRI alterations appears to be a dynamic process and there is debate regarding the correlation between presence of the eye of the tiger sign and clinical findings [47,49]. It has been shown that alterations may precede the development of clinical signs [50] (i.e. in asymptomatic carriers of homozygous mutations) but, on the other hand, may also be absent in early disease stages [43,47,51,52] or the bright spot may vanish over time [47]. In a recent study by Delgado *et al*. [47] only a proportion of patients had the typical eye of the tiger sign and four out of 20 patients did not have any T2-hyperintsity or T1-hypointensity. None of the heterozygous mutation carriers showed increased brain iron deposits [47]. These studies give further rise to the debate in how far iron may be causative or an epiphenomenon of neurodegeneration [53]. 

Using diffusion tensor imaging, increased fractional anisotropy along with abnormal mean diffusivity was demonstrated in GP and SN of patients compared to controls, probably due to iron deposits disturbing the local magnetic field [47,54]. Proton MR spectroscopy is not characteristic consistently showing markedly decreased *N*-acetylaspartate in the GP reflecting neuronal damage [55]. Dopamine transporter (DaT) SPECT imaging, a measure of striatal dopamine function, is generally normal in PKAN, [56,57] although abnormal findings have been reported [42] in line with the clinical experience that PKAN may manifest as parkinsonism [58-61] Cardiac 123I-meta-iodobenzylguanidine (MIBG) imaging which is used to assess postganglionic neuronal function of the sympathetic nervous system was also normal in PKAN, in contrast to PD and other Lewy body disorders where uptake is typically reduced [62]. Transcranial sonography [63,64] demonstrated bilateral hyperechogenicity in the SN and lenticular nucleus. It was thus suggested that transcranial sonography may be used as an inexpensive and simple screening method for the diagnosis of NBIA.

### Pathology in PKAN

Brown discoloration affecting the globus pallidus is seen on pathological assessment. A recent pathological study [65] in six genetically-proven cases revealed PKAN affects the CNS, whereas there is only occasional peripheral manifestation (including testicular pathology). Microscopic changes predominantly affected the GP with variable involvement of adjacent structures (medial putamen and internal capsule), while the cortex, brainstem and remaining deep grey nuclei were remarkably spared. Optic nerves and the cerebellum were not affected (in contrast to PLA2G6). Occasional intact neurons appeared in the GP, suggesting accumulation of abnormally ubiquitinated protein may precede other manifestations of degeneration and degenerative processes may affect the cytoplasm more than nuclear structures or neurons [65]. Two types of spheroids were recognized: larger, granular structures reflecting degenerating neurons and smaller, more intensely eosinophilic spheroidal structures representing swollen dystrophic axons. 

Iron accumulation was present in the GP, in a perivascular distribution, both as ferric iron (Fe^3+^; the paramagnetic form putatively associated with MRI hypointensity) but to a lesser degree also ferrous iron (Fe^2+^). “Iron dust”, a term used to describe diffuse iron-staining, of the neuropil was also seen. Macrophages were iron-loaded, as were astrocytes which strongly stained for ferritin. There was only faint tau expression and neurofibrillary tangles and tau-positive neurites were absent [65]. There was only minimal loss of neuromelanin in the SN, red nucleus and other brainstem areas which was compatible with normal aging. 

Numerous published reports have described Lewy body pathology in the brains of patients with NBIA [66-71]. However, in the recent series of gene proven-cases Lewy bodies were absent (in contrast to PLA2G6, see below). This is an important observation as this may mean that some of the historical “Hallervorden Spatz disease” cases *with* Lewy body pathology (published prior to gene identification) may in fact not have had PKAN but at least a proportion may have had NBIA type 2. We also suspect that historical reports in the literature lumped together various genetically-heterogeneous subtypes of iron accumulation disorders and that, in hindsight, the terms “Hallervorden Spatz disease”, “NBIA”, “PKAN” and others were used imprecisely. Interpretation of the older literature is thus problematic. However, even nowadays, imprecision of terminology continues and genetically-undetermined forms may be reported under the heading of “PKAN” rather than the wider umbrella term of NBIA. As this clouds the analysis of these disorders, precise use of terminology is essential [2,3].

### Genetic Testing and Molecular Findings in PKAN

Mutations, mostly missense, have been detected in all seven exons of the *PANK2 *gene. Deletions, duplications and splice-site mutations as well as exon deletions have also been reported [58]. Some mutations may be associated with milder phenotypes than others [35,43]. Two common mutations account for about one third of all PKAN cases, that is 1231G>A and 1253C>T. The majority of the remainder cases carry “private mutations” like the founder mutation, 680A>G, harboured by the patients from the Dominican Republic.

PANK2 is most prominently expressed in neurons of the cortex, GP, nucleus basalis of Meynert, and pontine nuclei. The exact pathophysiology of PKAN remains poorly understood. The associated *PANK2*-encoded protein governs the first regulatory step of coenzyme A synthesis by catalyzing the phosphorylation of pantothenate (vitamin B5) to yield phosphopantothenate [72]. Coenzyme A is essential for fatty acid synthesis and dysfunction of PANK2 thus likely causes derangement in lipid metabolism. PANK2 is mainly targeted to mitochondria, its mutation may therefore also cause dysfunction of cellular energy metabolism [73]. Indeed, a recent study examining blood metabolic profiles in PKAN documented elevated levels of lactate suggesting mitochondrial dysfunction and reduced levels of triglycerides, cholesterol metabolites and sphingomyelins confirming the role of PANK2 in the lipid metabolism [74]. It has been hypothesized that the alteration of ferroportin (FPN1) expression mediated by PANK2 might be the link to accumulation of iron in the brain [75]. 

### Treatment of PKAN

Treatment for NBIA disorders in general remains symptomatic. Stereotactic procedures, i.e. permanent lesioning or deep brain stimulation of several brain targets may produce some benefit (Table **4**) [76-79]. Although thalamotomy [80] and pallidotomy [81-83] were shown to be beneficial in individual patients with PKAN, irreversible lesioning procedures are used scarcely nowadays. The most effective practice appears to be deep brain stimulation of the posteroventral part of globus pallidus internus (DBS GPi). However, compared to primary generalized dystonias with most studies showing long-lasting 21-95% improvement in the Burke-Fahn-Marsden Dystonia Rating Scale (BFMDRS), the outcomes of DBS GPi in NBIA are more variable and at large less favourable [84-86]. In the largest series of 23 PKAN patients, at follow-up 9-15 months postoperatively, dystonia severity assessed by the BFMDRS had improved by 20% or more in two thirds of patients [87]. Although the extent of iron deposits and GP damage seem not to influence the immediate outcome of DBS GPi, benefit gradually diminishes over time due to disease progression. Some have recommended to operate on patients at an early stage in order to prevent fixed skeletal deformities and improve quality of life [87]. Others have suggested that a beneficial effect can be expected in patients with mobile and axial dystonia rather than fixed and oromandibular dystonia [88]. In patients with pharmacoresistant status dystonicus, pallidotomy or DBS GPi may be a life-saving intervention [52,82,89]. Interestingly, a recent study in seven patients showed that along with motor function cognitive abilities may be also improved after DBS GPi, possibly due to amelioration of attention distractibility caused by involuntary movements [90]. Thalamus and subthalamic area have been used as. Alternative targets for DBS in a small number of NBIA patients. Currently, there is only a few data comparing outcomes of different targets but they do not seem to have any advantage over GPi. 

Experimentally, 1-Hz repetitive transcranial magnetic stimulation of the premotor cortex produced mild temporary benefit in one case [91]. Turning to the underlying pathophysiology in drosophila models of PKAN supplementing pantethine restored CoA levels resulting in improved mitochondrial function, enhanced locomotor abilities and increased lifespan [92]. Thus, adding patethine to the food led to reduction of oxidative damage of proteins and improved larval crawling motor abilities in *dPANK/fbl* mutant compared to wild type drosophila flies. *PANK2* knockout mice have so far generally failed as a model for PKAN [93]. Results from human trials assessing a neuroprotective role of pantothenic acid (vitamin B5) are to our knowledge not yet available, but individual trials are disappointing (personal communication). For aspects on chelation therapy see below. 

## NBIA TYPE 2 – PLA2G6-ASSOCIATED NEURODEGENERATION (PLAN) 

The second core NBIA syndrome is PLAN due to *PLA2G6* gene mutations (NBIA type 2). Similar to PKAN there seems to be an age-dependent phenotype. Early-onset cases have infantile neuroaxonal dystrophy (INAD) characterized by progressive motor and mental retardation, marked truncal hypotonia, cerebellar ataxia, pyramidal signs, and early visual disturbances due to optic atrophy. Fast rhythms on EEG are frequently found and seizures may be present [94]. When onset of PLA2G6-associated neurodegeneration is later the phenotype may be atypical (atypical neuroaxonal dystrophy). We have encountered a case who presented with dystonia-parkinsonism combined with pyramidal signs, eye movement abnormalities, cognitive decline and psychiatric features [95]. Parkinsonism (the condition was subsequently assigned the PARK14 locus) was characterized by the presence of tremor including a pill-rolling rest component, rigidity and severe bradykinesia with a good response to levodopa in line with the finding of Lewy body pathology (see below). However, early development of dyskinesias is common, similar to other forms of early-onset parkinsonism with or without pyramidal signs [95,96]. Cerebellar signs and sensory abnormalities which are often prominent in the early childhood variant were absent. 

In line with this, neuroimaging shows cerebellar atrophy occurring in early stages of INAD, but not in late-onset disease. Although half of INAD patients may lack signs of iron accumulation early in the disease course [94], they usually develop hypointensity of the GP reflecting iron, noted on T2, T2* and proton density –weighted images [97]. Notably, the signal abnormality differs from the “eye of the tiger” sign of PKAN in that there is no central hyperintensity. Iron deposits in the SN are present in some atypical cases [98,99]. Contrary to PKAN, iron accumulation is not a universal feature of PLAN. The majority of late-onset cases lack signs of iron accumulation and MRI may even be completely normal. Others may show cortical atrophy or white matter changes. Thus, not all forms of *PLA2G6*-related neurodegeneration fall into the group of NBIA but there is “neuroradiological variability” [95]. PLAN should thus also be considered in patients with dystonia-parkinsonism even without increased brain iron on MRI [95]. 

The* PLA2G6* gene is located on chromosome 22q and contains 17 exons. The encoded protein, iPLA2 beta, is a group *via *calcium-independent phospholipase A2 that hydrolyzes the sn-2 acyl chain of phospholipids, thereby generating free fatty acids and lysophospholipids. iPLA2 beta is thought to play a role in remodeling of membrane phospholipids, signal transduction, cell proliferation, and apoptosis. It has been suggested that in case of loss of iPLA2 function lipid composition of the plasma membrane, vesicles, or endosomes may be altered. This may then affect proteins and processes normally involved in regulating the movement of membranes within axons and dendrites, subsequently leading to accumulation of membranes in distal axons, eventually culminating in progressive neurological impairment [100-102]. Recent functional phenotype-genotype studies [103] revealed that, compared to the wild type, mutant proteins associated with INAD exhibited less than 20% of the specific activity in both lysophospholipase and phospholipase assays, which predicted accumulation of PLA2G6 phospholipid substrates. In contrast, mutations associated with dystonia-parkinsonism did not impair catalytic activity, which may explain the relatively milder phenotype and absence of iron accumulation in at least some cases. It was hypothesized that mutations causing the dystonia-parkinsonism phenotype may be linked to abnormal regulation of PLA2G6 function and consequent activation of apoptotic pathways.

Pathologically, compared to PKAN in *PLA2G6*-associated neurodegeneration changes are more widely distributed throughout the CNS [104]. Early descriptions [28] of the pathological pattern noted cerebellar atrophy and sclerosis, accumulation of lipid and gliosis in the striatum, and degeneration of the optic pathway and of some of the long tracts in the brain-stem and spinal cord, i.e. the pyramidal, spinocerebellar, spinothalamic, and the gracile and cuneate fasciculi. In recent studies in both gene-proven mouse models [100] and human brains [99,105] widespread alpha-synuclein-positive Lewy pathology has been identified strengthening the link of PLA2G6 to idiopathic PD. Changes were particularly severe in the neocortex, corresponding to Braak stage 6 of the “diffuse neocortical type” of idiopathic PD [99]. In line with early clinical and imaging signs of cerebellar involvement, variable depletion of cerebellar cortical neurons (granular cells more than Purkinje cells) accompanied by marked astrocytosis was present [99]. Accumulation of hyperphosphorylated tau in both cellular processes as threads and neuronal perikarya as pretangles and neurofibrillary tangles, corresponding to Braak and Braak stage 5, has also been observed, again in contrast to PKAN [99]. Milder phenotypes in late-onset disease tended to show less tau involvement [106,107]. 

## FA2H-ASSOCIATED NEURODEGENERATION (FAHN)/ SPG35

In two consanguinous families from Italy and Albania* FA2H* mutations were recently identified as another cause of NBIA (Table **2A**) [106]. The gene is also associated with leukodystrophy [108] and a form of hereditary spastic paraplegia (HSP) [109] and overlapping syndromes [110,111]. FA2H is thus another example of how alterations in a distinct gene produce phenotypes that are much wider than originally anticipated.

The clinical phenotype was characterized by childhood-onset gait impairment, spastic quadriparesis, severe ataxia and dystonia. Seizures and divergent strabismus may also be present. Overall there was great similarity between the clinical presentations of neuroaxonal dystrophies. MRI demonstrated bilateral GP T2 hypointensity, consistent with iron deposition, prominent pontocerebellar atrophy, mild cortical atrophy, white matter lesions and corpus callosum thinning.

Like PANK2 and PLA2G6, the metabolic pathway of FA2H involves the lipid and ceramide metabolism [112]. FA2H catalyzes hydroxylation at position 2 of the N-acyl chain of the ceramide moiety. Glycosphingolipids which contain a high proportion of 2-hydroxy fatty acid are important constituents of myelin sheaths [109]. In turn, FA2H deficiency results in abnormal myelin, giving rise to the allelic disorders leukodystrophy and the HSP subform SPG35. Between these clinical entities radiological overlap has been noted. White matter changes are present in FAHN and are also a core element of leukodystrophies. Presence of a thin corpus callosum seen in FAHN is also a hallmark feature in some of the HSPs [113]. 

Notably, a link between HSP and dystonia-parkinsonism was recently also described for other HSP subtypes, i.e. SPG11, SPG15 and genetically undetermined HSP forms, [114-117] and Lewy body pathology has been present in individual HSPs cases with parkinsonism, with or without dystonia [117,118]. 

Mouse models of FA2H have recently been developed [119,120]. In these, marked demyelination and profound axonal loss in the CNS could be demonstrated after a period of normal myelin development [119,120]. Axons were abnormally enlarged and there was abnormal cerebellar histology. In contrast, structure and function of peripheral nerves were largely unaffected. Pathological studies of human FAHN brains are not yet available. 

## MITOCHONDRIAL MEMBRANE PROTEIN ASSOCIATED NEURODEGENERATION (MPAN)

Hartig *et al*. [121] recently described a cohort of Polish NBIA patients including a subgroup of 24 cases with childhood-onset dysarthria and gait difficulty, followed by the development of spastic paraparesis, extrapyramidal features (dystonia and parkinsonism), neuropathy, optic atrophy and psychiatric symptoms. Iron deposition was present in the globus pallidus and substantia nigra. A similar case presenting with progressive tremor, dystonia and spasticity, as well as peripheral neuropathy, optic atrophy, and cognitive decline has been reported [122]. 

Genetic work-up led to identification of the new NBIA gene, *C19orf12*, at chromosome 19q12 [121]. (Table **2B**) A deletion of eleven basepairs leading to a premature stop codon and predicted to cause early truncation of the protein was identified in the majority of patients due to a founder effect in the Polish cohort. Notably, one patient with a different *C19orf12* mutation had a milder phenotype resembling idiopathic PD. 

Post mortem brain examination MPAn revealed iron-containing deposits in the GP and SN, axonal spheroids, Lewy body-like inclusions and tau-positive inclusions in various regions of the brain [121]. Little is known about gene function, but it is localized predominantly in mitochondria and it is co-regulated with genes involved in fatty acid metabolism. Thus, it is possible that *C19orf12* gene maps to the same metabolic pathway as *PANK2* and *PLA2G6*. The *C19orf12* gene abnormality should not be confused with disorders associated with mutation in the *C9orf12* gene, located on chromosome 9 and associated with familial fronto-temporal dementia and motor neuron disease [123-125].

## KUFOR-RAKEB DISEASE (PARK9)

Kufor-Rakeb disease is a rare autosomal recessive neurodegenerative disease originally described in a consanguineous Jordanian family [126] from the village of Kufor-Rakeb. The associated gene was later identified in a large Chilean sibship, [127] and since then other cases have been identified from various countries and carrying different mutations (Table **2C**). The clinical phenotype of Kufor-Rakeb disease comprises parkinsonism, with pyramidal tract signs in some. Eye movement abnormalities with incomplete supra-nuclear upgaze palsy can be a clue. Slowing of vertical and horizontal saccades and saccadic pursuit have also been described [128]. Oculogyric dystonic spasms, facial-faucial-finger mini-myoclonus and autonomic dysfunction may be present. Psychiatric features include visual hallucinations and dementia. Disease onset is usually in adolescence [126, 127,129-131]. A good response to levodopa has been noted; [126] however, similar to other complicated recessive dystonia-parkinsonism variants, levodopa-induced dyskinesias tend to develop early [129,130]. Brain imaging may show diffuse moderate generalized atrophy. Iron deposition within the basal ganglia affecting the putamen and caudate is present in some (Fig. **1A**), including one of our cases and the Chilean family mentioned above, [132,133] although not all [129,130,134]. On transcranial sonography the substantia nigra was found to be normal [133], in contrast to PKAN and idiopathic PD where hyperechogenicity can usually be detected. Dopamine transporter imaging showed marked bilateral symmetrical reduction of striatal activity indicative of diminished presynaptic activity [133]. Electrophysiological studies suggested pyramidal tract damage, in line with clinical findings [135]. Motor evoked potential (MEP) latencies were increased in patients, but Subtle electrophysiological abnormalities were also present in asymptomatic heterozygous *ATP13A2* mutation carriers [135]. 

Kufor-Rakeb disease is due to mutations in the *ATP13A2 *[127] gene located on chromosome 1p. The 26 kb-spanning gene contains 29 exons and encodes a lysosomal 5 P-type ATPase. Most patients reported to date carried homozygous mutations (Table **2B**) but compound heterozygous cases have also been identified. 

Sural nerve biopsy [136] has shown acute axonal degeneration, some regeneration, and a very mild chronic inflammatory response with endoneurial and epineurial T-cells. Within Schwann cells, perineurial and epineurial cells, but not within axons, numerous cytoplasmic inclusion bodies were seen. Electron microscopy revealed the inclusions to be membrane-bound, irregular, and occasionally folded. Overall they resembled irregular primary lysosomes [136]. The role of the lysosome is supported by functional studies which showed premature degradation of mutant ATP13A2 proteins by the proteasomal, but not the lysosomal pathways [137]. More recent functional studies strengthened the role of ATP13A2 for mitochondrial renewal and maintenance when decreased autophagy was observed in ATP13A2-deficicent cells which led to increase of mitochondrial mass, secondarily affected mitochondrial quality control and resulted in increased ROS production [138]. In fibroblasts from patients impaired mitochondrial clearance was detected [139] with a higher frequency of mitochondrial DNA lesions, increased oxygen consumption rates, and increased fragmentation of the mitochondrial network. Overexpression of wild-type ATP13A2, however, rescued the respiration phenotype. The mechanism of iron accumulation in Kufor-Rakeb disease is unclear and probably different form the above mentioned disorders. There is increased awareness of lysosomal role in iron metabolism and recycling, [140] it is thus conceivable that lysosomal dysfunction could alter reuse of “old” cellular iron and lead to its increased uptake. 

While brain pathology is not available from any patient diagnosed with Kufor-Rakeb disease during life, genetic work-up using exome sequencing recently allowed retrospective identification of *ATP13A2* mutations in a family diagnosed with juvenile neuronal ceroid-lipofuscinosis (NCL) [141-143] for who brain pathology is available [144]. The clinical phenotype was characterized by progressive spinocerebellar ataxia, bulbar syndrome, extrapyramidal and pyramidal involvement and intellectual deterioration. Post-mortem pathological examination showed abundant neuronal and glial lipofuscinosis involving cortex, basal nuclei, cerebellum, but sparing the white matter. Whorled lamellar inclusions were typical of NCL in electron microscopy. Lipofuscin deposits were confirmed in the retina. Muscle biopsy showed numerous subsarcolemmal autofluorescence bodies with a fingerprint appearance in electron microscopy and suggestion of neurogenic muscular atrophy. One may speculate in how far ATP13A2 mutations may also explain other NCL cases, however reports predated the genetic era [145,146].

A similar link between NCL and Kufor-Rakeb disease was established when *ATP13A2* mutations were recently identified in Tibetean terriers with NCL [147,148]. In this context it is interesting that some patients with NCL have parkinsonism and that brains of NCL patients caused by Cathepsin D deficiency (CLN10) show intense alpha-synuclein staining [144]. 

## ACERULOPLASMINEMIA

**Fig. (2):**
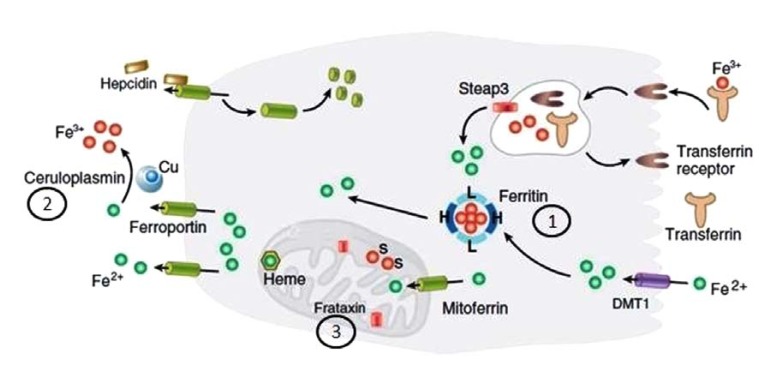
Pathways of cellular iron homeostasis and neurological disorders associated with iron accumulation associated with these, adjusted from *Madsen and Gitlin* [192] and reproduced from [3]. Iron uptake occurs *via* the divalent transporter DMT1 (ferrous iron, Fe 2+, shown in the lower right of the figure)or *via* endocytosis of the transferrin receptor (ferric iron, Fe 3+, shown in the upper right of figure). Steap3 is a ferrireductase critical for transferrin-mediated iron release into the cell. Ferritin is the predominant storage protein consisting of heavy chains and light chains. Mutations in the gene encoding ferritin light chains are associated with neuroferritinopathy (1). Iron homeostasis is regulated by hepcidin which binds to ferroportin, the only known cellular iron exporter. Ceruloplasmin is a ferroxidase mediating efficient cellular iron release. Mutations in the gene encoding ceruloplasmin cause aceruloplasminemia (2). Iron enters mitochondria *via* mitoferrin, Frataxin is a mitochondrial protein mediating Fe-S cluster formation and heme biosynthesis. Mutations in *frataxin* cause Friedreich ataxia (3).

Aceruloplasminemia is due to mutations in the *ceruloplasmin gene *on chromosome 3q in which more than 40 mutations have been described. (Tables **1** and **3**, Fig. **2**) Inheritance is autosomal recessive. Most reported patients hail from Japan with an estimated prevalence of 1:2,000,000, but several patients from China, America and Europe have been reported. The clinical presentation is characterized by adult-onset movement disorders and dementia. A recent literature review [149] revealed an average age at diagnosis of 51, ranging from 16 to 71 years. For the 28 homozygous cases [149] the most common presenting feature was cognitive impairment (42%) accompanied by craniofacial dyskinesia (28%), cerebellar ataxia (46%) and retinal degeneration (75%), which may histopathologically resemble age-related macular degeneration [150]. Diabetes mellitus and microcytic anemia may be associated and frequently predate neurologic symptoms. General fatigue and chronic asthenia are also commonly reported complaints.

The encoded protein plays a crucial role in the mobilization of iron from tissues through its ferroxidase activity and carries 95% of the plasma copper. Protein dysfunction results in excessive iron accumulation not only in the brain (basal ganglia, thalami, dentate nuclei and cerebral and cerebellar cortices) but also within the retina, pancreas and liver. The profound cortical involvement has not been reported in other NBIAs and probably underlie the high prevalence of cognitive dysfunction. Autopsy findings include mild degree of cortical atrophy, large iron deposits in basal ganglia, thalami, dentate nuclei and cerebral cortices predominantly in the perivascular spaces localized mostly to terminal astrocytic processes and deformed astrocytes with swollen, oxidatively damaged astrocytic foot processes appearing as globular structures [151-153]. These results suggest that astrocytes, which are necessary for brain iron uptake, detoxification and further trafficking, bear the brunt of the disease. Decreased activity of mitochondrial respiratory chain complexes I and IV and elevated markers of lipid peroxidation were also described in autopsied brains [154]. Overall, there is good evidence that enhanced oxidative stress caused by redox active iron is a major cause of neurodegeneration in aceruloplasminemia [152,153,155]. Neuronal cell death may be partially secondary to the loss of protective function normally provided by astrocytes. Although aceruloplasminemia is considered autosomal recessive, mildly increased iron loads in liver and basal ganglia as well as neurologic symptoms have been described also in heterozygotes [59,156-158]. Diagnostically in homozygotes, ceruloplasmin is typically undetectable in the serum, and copper and iron serum levels are low. Ferritin on the other hand is elevated 3–40-fold [149]. Hypometabolism in the basal ganglia and the thalamus has been detected on FDG-PET [154].

## NEUROFERRITINOPATHY

Mutations in the *FTL* gene on chromosome 19q cause neuroferritinopathy (also called hereditary ferritinopathy). To date at least seven pathogenic mutations have been reported including six frameshift mutations and one missense mutation (Table **2D**). The former alter the reading frame and are predicted to lead to an extended peptide at the site of the pore in the ferritin molecule [159]. Of these, an insertion at position 460 accounts for most cases due to a founder effect. This most common mutation, c.460InsA, is clustered in the region of Cumbria in England due to a founder effect. Independent cases carrying private mutations were reported from France, Canada and Japan [160-164]. Interestingly, ferritin inclusions were found beside CNS also in the skin, muscle, kidney and liver in a large French pedigree carrying mutation 498-499InsTC [165]. Inheritance is autosomal dominant and neuroferritinopathy is thus the only NBIA syndrome with dominant inheritance. 

Mean onset age is in midlife, around age 40, with extrapyramidal features including chorea, stereotypies, and dystonia with phenotypic similarity to Huntington’s disease or neuroacanthocytosis [161]. Fairly typical symptom is orolingual-mandibular dyskinesia associated with jaw dystonia and blepharospasm during phonation producing dysarthria and tongue biting. About 10 percent present with parkinsonism. Pyramidal involvement and ataxia are usually absent, however cerebellar symptoms and tremor were described in one family [165,166]. Cognitive dysfunction, depression and psychosis may be present. 

In contrast to aceruloplasminemia, serum ferritin concentration may be low. MRI may reveal cystic changes in the basal ganglia and bilateral pallidal necrosis, in addition to iron accumulation in the caudate, GP, putamen, SN, and red nuclei [59]. Even in the asymptomatic phase of the disease hypointense signals suggestive of early iron accumulation were present, as shown in three gene mutation carriers [159]. The severity of T2* abnormality increased with age. The authors concluded that iron deposition in neuroferritinopathy actually begins in childhood but the disease usually does not become symptomatic until midlife. This has implications for timing of the chelating therapy which should be optimally started in childhood in order to prevent iron accumulation. Chorea and stereotypy associated with neuroferritinopathy may respond well to tetrabenazine, a monoamine depletor [143,167]. 

Pathology [168] has revealed ferritin-positive spherical inclusions ferritin inclusions localized extracellularly and intracellularly in iron-rich areas, often co-localizing with microglia, oligodendrocytes, and neurons. Neuroaxonal spheroids immunoreactive to ubiquitin and tau, and neurofilaments have been reported, bridging the gap to the group of neuroaxonal dystrophies discussed above. The main sites of involvement are the posterior putamen and cerebellum, but extracerebral pathology such as hepatic iron deposits may be present in some patients [169]. A recently developed mouse model confirmed the build-up of iron in the brain reminiscent of the human disease and suggested a key role of toxic ferritin aggregates and oxidative damage to mitochondria in the pathogenesis [170,171]. Studies with cellular cultures confirm that oxidative damage is the primary cause of cellular degeneration in neuroferritinopathy [172,173].

## SENDA SYNDROME AND OTHER NBIA SYNDROMES INCLUDING GENETICALLY YET UNDETERMINED FORMS

A genetically yet undetermined form has recently been described under the umbrella term of “static encephalopathy (of childhood) with neurodegeneration in adulthood” (SENDA syndrome) [174]. The clinical phenotype consisted of early-onset spastic paraplegia and mental retardation which remained static until the late 20s to early 30s but then progressed to parkinsonism and dystonia. Additional features included eye movement abnormalities, sleep disorders, frontal release signs and dysautonomia. Imaging showed brain iron accumulation affecting the GP and hypointensities in the SN, as well as white matter changes. Therapeutically, there was a marked response to levodopa in those in whom it was tried. No genetic cause has yet been identified. 

In addition there remain single case reports of patients with NBIA including late-onset cases with a parkinsonian phenotype resembling Parkinson’s disease [43,175]. Rest tremor was asymmetric with a re-emergent component. Levodopa treatment led to development of dyskinesias, but deep brain stimulation was of good benefit. 

Finally, brain iron accumulation also occurs in other conditions, such as Friedreich’s ataxia (Fig. **2**), DRPLA, Woodhouse–Sakati syndrome, mannosidosis, fucosidosis, mucolipidosis type IV superficial siderosis and others, but, with different areas of highest iron accumulation density [2,176,177]. Iron dysregulation also plays a role in restless legs syndrome [178]. A classification based on the presumed mechanism has been proposed [2].

Finally, MRI iron deposition, sometimes resembling the eye of the tiger sign, has occasionally been observed in other neurodegenerative diseases [179-182]. 

## TREATMENT OF NBIA AND RELATED DISORDERS

Understanding the pathogenesis of NBIA and related disorder is critical in the development of mechanistic treatments. To date the therapeutic options for NBIA disorders remain largerly symptomatic. Pharmacotherapy, such as dopaminergic drugs, anticholinergics, tetrabenazine and other drugs may bring some relief, but they rarely satisfactory and have no impact on the long-term outcome. Deep brain stimulation can produce some benefit (Table **4**) but does not halt neurodegeneration. With the assumption that iron plays a causative role, chelators which reduce the amount of free iron are being explored [183,184]. Promising animal models initiated trials in humans and single cases with beneficial effects have been reported, however with mixed results so far (Table **5**). Most published case studies were performed on aceruloplasminemia patients. Chelating therapy generally proved to be capable of decreasing the amount of brain iron as assessed by quantitative MRI methods. Despite this, clinical benefit was not observed in some of these patients. It is unclear why some patients are non-responders while other patients are, but it was suggested that chelating therapy should be tried in aceruloplasminemia. Interestingly, there are three reports on chelating treatment in idiopathic NBIA patients with clinical benefit. This suggests that unknown diagnosis may not necessarily prevent initiation of chelating treatment [185-187]. In addition to individual case reports, results of the first phase II pilot open trial in PKAN have been recently published, assessing the clinical and radiological effects of the oral iron-chelator deferiprone at a dose of 25 mg/kg/day over a 6-month period [188]. Of nine patients who completed the study, six had classic and three had atypical disease. Median disease duration was 11 years. Deferiprone was overall well tolerated. Side effects included nausea and gastralgia (44%) but no serious adverse event occurred. The authors observed a significant (median 30%) reduction in GP iron content, ranging from 15-61%. However, there was no clinical benefit, as rated on the Burke-Fahn-Marsden Rating Scale and SF-36 scale which may be due to the relatively short treatment duration or already long disease duration neuronal damage too advanced to allow a rescue of function. In how far early treatment, i.e. in asymptomatic mutation carriers, can prevent or delay the development of neurological symptoms is unknown, but was of little benefit in the case of neuroferritinopathy [161]. In another study with 6 NBIA patients, 12-months deferiprone treatment was effective in 2 out of 4 PKAN and one of two idiopathic NBIA patients [186]. Further studies to evaluate the efficacy of chelating therapy are needed.

## REMARKS, THOUGHTS AND CONCLUSIONS

We have summarized the aspects of iron metabolism both in the physiological and pathological state and reviewed NBIA disorders including major forms as well as clinically characterized subgroups of yet undetermined genetic etiology. The common theme is that the various NBIA syndromes are characterized by remarkable clinical and genetic heterogeneity. FA2H for example demonstrates how mutations in a single gene can give rise to numerous different disease manifestations. For PKAN and PLAN an age-dependent phenotype has been recognized. In time it is likely that yet wider phenotypes will emerge for the core syndromes. Furthermore, we expect that new genes underlying NBIA will be discovered, which may map into the pathways [112] of iron (also see Dusek *et al*. [2], Table **1**), lysosome and/or ceramide metabolism Fig. (**3**). However, any search for a genetic cause still relies on meticulous clinical characterization. We, therefore, encourage our colleagues to look out for and report their NBIA cases, providing as much detail about the history, phenotype (preferably documented by a video), natural history, response to treatment and any unique aspects of the disease. Furthermore, while new gene products of yet undetermined forms may lie on related biochemical routes, we may also discover that other genes involved in yet uncharacterized related pathways may be associated with similar syndromes [53,95]. 

These pathways may bridge the gap to yet new conundrums of disorders and key players connecting these may be identified. Currently, the focus is on the lysosomal function and ceramide, a central molecule in sphingolipid metabolism composed of an N-acylated sphingosine. Ceramide is metabolized in lysosomes and is involved in many cellular processes (for review see [189,190]) including Lewy body pathology and tauopathies and is thereby linked to the common idiopathic neurodegenerative diseases including PD, other parkinsonian syndromes, and possibly Alzheimer’s disease [30,106,112]. However, despite recent advance, whether iron accumulation is causative or a consequence still remains a matter of debate and will likely depend on the particular disease. 

## Figures and Tables

**Fig. (3):**
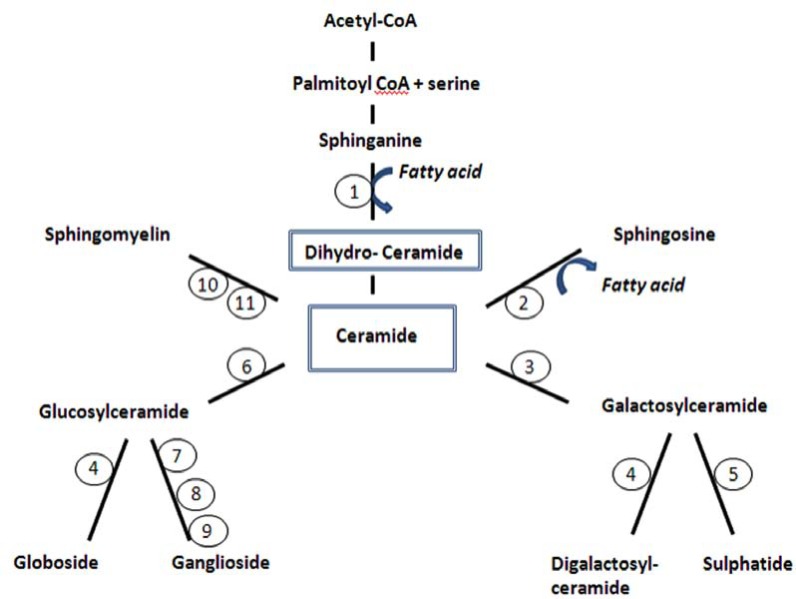
The figure shows simplified metabolic pathways of ceramide which is derived from two main sources operating in different cellular compartments: hydrolysis of membrane-derived sphingomylin and de novo synthesis from palmitoyl CoA and serine. Neurological diseases along the pathways are demonstrated (incomplete list). (for reference see King 2008 [203] and http://www.sphingomap.org/) 1 - Pantothenate kinase associated neurodegeneration (PKAN), 2 - Farber disease (ceraminidase deficiency), 3- Krabbe disease (beta galactosidase deficiency), 4 - Fabry disease (alpha galactosidase A deficiency), 5 - Metachromatic leukodystrophy (cerebroside sulphatase deficiency), 6 - Gaucher disease (glucocerebroisidase deficicency), 7 - Tay Sachs disease (hexosaminidase A deficiency), 8 - GM2 gangliosidosis (beta-galactosidase deficiency), 9 - Sandhoff disease (hexosaminidase A + B deficiency), 10 - PLA2G6-associated neurodegeneration, 11 - Nieman Pick disease (sphingomyelinase deficiency). Reproduced from Schneider *et al*. [3].

**Table 1.** Overview of NBIA Conditions and Genes (if Known)

Condition (Acronym)	Synonym	Gene	Chromosomal Position	Areas of Highest Iron Density	Reports of Gene-proven (Symptomatic) Cases without Iron	Reports of Presymtomatic Cases with Iron
PKAN	NBIA1	*PANK2*	20p13	GP, MRI eye of the tiger sign (central hyperintensity within a surrounding area of hypointensity)	Yes	Yes
PLAN	NBIA2, PARK14	*PLA2G6*	22q12	GP. Additional SN involvement in some	Yes	No
FAHN	SPG35	*FA2H*	16q23	GP. Often white matter changes.	Yes	No
MPAN	--	C19orf12	19q12	GP and SN.	No	No
Kufor-Rakeb disease	PARK9	*ATP13A2*	1p36	Putamen and caudate.	Yes	No
Aceruloplasminemia	--	*CP*	3q23	Basal ganglia, thalamus, dentate nuclei and cerebral and cerebellar cortices. Liver, pancreas.	Yes*	Yes
Neuroferritinopathy	--	*FTL*	19q13	Caudate, globus pallidus, putamen, substantia nigra, and red nuclei.	Yes	Yes
SENDA syndrome	--	n.k.	n.k.	GP and SN. White matter changes	n/a	n/a
Idiopathic late-onset cases	--	Probably heterogeneous	Probably heterogeneous	Heterogeneous.	n/a	n/a

CP = ceruloplasmin, FA2H = fatty acid 2-hydroxylase, FTL = ferritin light chain, GP – globus pallidus, MPAN – mitochondrial membrane-associated neurodegeneration; NBIA = Neurodegeneration with brain iron accumulation, PANK2 = Pantothenate kinase 2, PKAN = pantothenate kinase-associated neurodegeneration, PLA2G6 = phospholipase A2, PLAN = PLA2G2-associated neurodegeneration, SENDA = static encephalopathy of childhood with neurodegeneration in adulthood, SENDA = static encephalopathy (of childhood) with neurodegeneration in adulthood; SN = substantia nigra, SPG = spastic paraplegia. n.k. = not known For other syndromes that may be associated with iron accumulation, see [2].

* Skidmore *et al*. 2008 [191] present a case of suspect ACP just with cerebral atrophy. However, genetic examination was not performed.

**A):** Reported Mutations of FAHN

Nucleotide Change	Amino Acid Change	References
c.703C>T	p.Arg235Cys	Dick *et al* 2010
c.157_174del18	p.Arg53_Ile58del	Dick *et al* 2010
c.786+1G>A	p.Glu205_Ser346del	Edvardson * et al* 2008
c.460C>T	p.Arg154Cys	Kruer *et al*. 2010
c.509_510delAC	p.Tyr170X	Kruer *et al*. 2010
c.270+3A>T	p.Gly91ValfsX43	Garone *et al* 2011
c.707T>C	p.Phe236Ser	Pierson * et al*. 2012

**B):** Reported Mutations of MPAN

Nucleotide Change	Amino Acid Change	References
c.32C>T	p.Thr11Met	Hartig *et al*. 2011
c.157G>A	p.Gly53Arg	
c.194G>A	p.Gly65Glu	
c.204_214del11	p.Gly69ArgfsX10	
c.424A>G	p.Lys142Glu	
c.362T>A	p.Leu121Gln	Horvath *et al*. 2012

**C):** Reported Kufor Rakeb Cases Carrying Homozygous or Compound Heterozygous Cases to Date

Country of Origin	Zygosity	Nucleotide Change	Amino Acid Change	References
Jordanian	Homozygous	c.1632_1653dup22	p.Leu552fs	Ramirez *et al*., Nature 2006
Chilean	Compound heterozygous	c.1306+5G>A, c.3057delC	Ex13skipping/fs p.G1019fs	Ramirez *et al*., Nature 2006; Brüggemann *et al*., Arch Neurol 2010
Brazilian	Homozygous	c.1510G>C	p.Gly504Arg	Di Fonzo *et al*., Neurology 2007; Chien *et al*., Mov Disord 2011
Japanese	Homozygous	c.546C>A	p.Phe182Leu	Ning YP *et al*., Neurology 2008
Pakistan	Homozygous	c.1103_1104insGA	p.Thr367fs	Schneider SA *et al*., Mov Disord 2010; Paisán-Ruiz *et al*., Mov Disord 2010
Afghan	Homozygous	c.2742_2743delTT	p.Phe851fs	Crosiers *et al*., Parkinsonism Relat Disord 2010
Italian	Homozygous	c.2629G>A	p.Gly877Arg	Santoro *et al*.; Neurogenetics 2010
Asian	Compound heterozygous	c.3176T>G and c.3253delC	p.L1059R, p.L1085WfsX1088	Park *et al*., Hum Mutat. 2011
Inuit	Homozygous	c.2473C>AA	p.Leu825fs	Eiberg *et al*. Clin. Genetics. 2011
Italian (Campania region)	Homozygous	c.G2629A	p.G877R	Santoro *et al*. Neurogenetics. 2011
Belgium*	Homozygous	c.T2429G	p.Met810Arg	Bras *et al*. Hum Mol Gen 2012

* clinically diagnosed with NCL

**D):** Cases of Neuroferritinopathy

Country of Origin	Nucleotide Change	Amino Acid Change	References
Japan	c.439_442dupGACC	p.His148ArgfsX34	Kubota *et al*. 2009
French Canadian and Dutch ancestry	c.442dupC	p.His148ProfsX33	Mancuso *et al.* 2005
France	c.458dupA	p.His153GlnfsX28	Caparros-Lefebvre *et al*. 1997; Devos *et al*. 2009
England, Cumbria and in one American family of German ancestry	c.460dupA	p.Arg154LysfsX27	Curtis *et al.* 2001; Chinnery *et al.* 2007; Ondo *et al*. 2010
Japan	c.469_484dup16nt	p.Leu162ArgfsX185	Ohta *et al*. 2008
Spanish-Portuguese Gypsy Origin	c.474G>A	p.Ala96Thr	Maciel *et al.* 2005
French Canadian	c.498insTC	p.Phe167SerfsX26	Vidal *et al*. 2004

**Table 3.** Comparison of Aceruloplasminemia and Neuroferritinopathy

	Aceruloplasminemia	Neuroferritinopathy
Gene	Ceruloplasmin gene	Ferritin light chain gene
Pattern on Inheritance	Autosomal recessive	Autosomal dominant
Presentation	Third decade—diabetes, anemia Fifth decade—neurologic	Third through sixth decade
Defect	Brain iron recycling	Brain iron storage
Pathogenesis	Brain iron accumulation Systemic iron accumulation in all	Brain iron accumulation Systemic iron accumulation in some
Clinical	Diabetes, anemia, dementia Dystonia, dysarthria	Dementia, dystonia, dysarthria
Pathology	Iron accumulation in astrocytes Neuronal loss	Iron accumulation in astrocytes Neuronal loss

Modified from Madsen and Gitlin [192].

**Table 4.** Reported Cases of Brain Lesioning and Deep Brain Stimulation in NBIA Disorders

Diagnosis Made by the Authors	Diagnostic Characteristics	Number of Patients	Age at Operation (Years)	Intervention	Result	References
Hallervorden Spatz disease	Eye of the Tiger sign	1	10	Unilateral pallidotomy	Functional improvement	Justesen *et al*. 1999
Hallervorden-Spatz disease with Status dystonicus		1	9	Bilateral pallidotomy	Alleviation of status dystonicus achieved in combination with temporary intrathecal baclofen infusions	Kyriagis *et al*. 2004 [82]
Hallervorden Spatz disease		1	10	Bilateral pallidothalamotomy	Improvement of BFM and Dystonia Disability Rating Scale (from 116 and 30 points to 41 and 18 points). Painful dystonia was resolved	Balas *et al*. 2006 [81]
Hallervorden Spatz disease		1	10	Bilateral thalamotomy	No clinical progression at 21 months from the last operation	Tsukamoto *et al*. 1992 [80]
Hallervorden Spatz disease		2	18, 20	DBS of the posterior part of the ventral lateral thalamic nuclei	BFMD scores are presented only in 1 case with a follow-up time of 120 months and an improvement of 26%.	Vercueil *et al* 2001 [193]
NBIA	Not genetically tested	1	36	Pallidal DBS	Improvement of BFMD of 80% at 1-year follow-up	Umemura *et al*. 2004 [194]
Hallervorden Spatz disease		3	n.k.	STN-DBS	High frequency STN-DBS had no effect on generalized dystonia	Detante *et al* 2004 [195]
PKAN	Genetically confirmed (1442del3 and 1583C_T)	1	13	GPi-DBS, frequency = 130 Hz, pulse width = 210_s, amplitude = 2.6 V.	Improvement of BFM (from 92 to 30 points) and BFMDS Disability Score (from 24 to 11), but then deterioratation until the final 5-year visit.	Krause *et al*. 2006 [78]
PKAN	Genetically confirmed	6	Mean 21 (range, 10-39)	Bilateral GPi-DBS	Motor improvement (range 46% to 91.5%), stable throughout the follow-up period (from 6 to 42 months).	Castelnau *et al*. 2005 [76]
Hallervorden Spatz disease	Eye of the Tiger sign	1	8	Bilateral GPi-DBS	Postoperatively, severe stridor preventing extubatation, tracheostomy. Subsequently, pneumonia. Death three months after the procedure	Sharma *et al*. 2005 [196]
PKAN	Eye of the Tiger sign	1	17	Pallidal DBS, frequency = 185 Hz, pulse width = 240 µs amplitude = 3.4 V	Improvement in BFMDRS-M at 2–6 months was 27.2% (from 86 to 66 points)	Shields *et al*. 2007 [197]
PKAN		1	16	Pallidal stimulation	Physical and psychosocial functioning improved	Isaac *et al*. 2008 [198]
PKAN	Genetically confirmed (A382V)	1	11	Pallidal DBS	Beneficial	Mikati *et al*. 2009 [77]
NBIA	Eye of the tiger sign in all and fourteen of them genetically confirmed PKAN. PANK2 mutations excluded in one	23	Mean 18 (range, 6-36)	GPi-DBS, frequency = 128-133 Hz, pulse width = 194-244µs, amplitude = 2.7-2.8 V.	At follow-up 9-15 months postoperatively improvement of dystonia by 20% or more in two thirds of patients	Timmermann *et al* 2010 [87] #
PKAN	Genetically confirmed (C1021T)	1	19	Bilateral GPi- DBS	Improvement of BFMDRS from 96 to 10 points	Grandas *et al*. 2011 [52]
PKAN	Genetically confirmed	4	n.d.	Pallidal DBS, frequency =60-130 Hz, pulse width = 60-120 µs, amplitude = 0.7-4.5 V,	Favorable in two patients with atypical PKAN with moderately severe dystonia (BFMDRS 44.5/38 and 46/39) and one patient with typical PKAN and status dystonicus (BFMDRS 96/74.5). However, minimal response in a patient with typical PKAN and severe symptoms (bedridden, limb deformity and multiple contractures, BFMDRS 79.5/80)	Lim *et al*. 2011 [89]
Idiopathic NBIA with parkinsonian phenotype		1	n.d.	STN DBS	Beneficial	Aggarwal *et al*. 2010 [43]
NBIA1	Diagnosis based on MRI. Previous unsuccessful ablation surgery	1	16	Bilateral STN stimulation	Improvement of BFMDRS from 114 to 35 (69% improvement) post-op. At follow-up 3 months: 28 points (75% improvement). 12 months: 14 points (88% improvement), 3 years after surgery: 84% improvement	Ge *et al*. 2011 [199]
PKAN	Genetically confirmed	2	17 and 16	Bilateral GPi- DBS frequency = 130 Hz, pulse width = 450µs, amplitude = 1.7 and 2.0 V.	BFMDRS 77.5 and 72 points preoperatively, 15 and 42.5 points 3 months postoperatively, 39 and 52 points 48 months postoperatively	Adamovicova *et al*. 2011 [88]
PKAN	Genetically confirmed	7	Mean 11.6 (range, 8-17)	Bilateral GPi- DBS	Improvement in BFMDRS and cognitive abilities in 6/7 patients assessed by subtests from age-appropriate Wechsler Intelligence Scale measuring non-verbal and verbal intellectual abilities and memory	Mahoney *et al*. 2011 [90]

BFMDRS = Burke-Fahn-Marsden Dystonia Rating Scale. n.d. = no details known,

# = partially cited from Krause *et al*. (2006), Shields *et al*. (2007), Umemura *et al*. (2004) and Kurlemann *et al*. (1991), Adamovicova *et al*. (2011) reports further observation in 2 patients from this cohort.

**Table 5.** Comparison of the Main Available Iron Chelators.

	Deferoxamine	Deferiprone	Deferasirox
Route of administration	Parenteral, usually subcutaneous or intravenous	Oral	Oral
Plasma half-life	Short (minutes); requires constant delivery	Moderate (< 2 hours). Requires at least 3-times per-day dosing	Long, 8–16 hours; remains in plasma at 24 h
Important side effects	Auditory and retinal toxicity; effects on bones and growth; potential lung toxicity, all at high doses; local skin reactions at infusion sites	Rare but severe agranulocytosis; mild neutropenia; common abdominal discomfort; erosive arthritis	Abdominal discomfort; rash or mild diarrhoea upon initiation of therapy; mild increased creatinine level
Ability to chelate intracellular cardiac and other tissue iron in humans	Probably lower than deferiprone and deferasirox (it is not clear why)	High in clinical and in *in vitro* studies	Insufficient clinical data available; promising in laboratory studies
Reported use in patients NBIA disorders*	Pan *et al*. (2011), aceruloplasminemia, n=1, 20-30% T2* increase in caudate/ SN, no clinical improvement	Zorzi *et al*. (2011), PKAN, n=9, median 30% T2* increase in GP, no clinical improvement	Finkenstedt *et al*. (2010), aceruloplasminemia, n= 2, no radiological or clinical change
	Hida *et al*. (2010), aceruloplasminemia, n= 1, no radiological, mild clinical change	Abbruzzese *et al*. (2011), PKAN (n =4) and idiopathic NBIA (n=2), 20-30% T2* increase in GP in 3 pts, Mild-moderate clinical improvement in 2 pts. Moderate clinical improvement in 1 pt	Skidmore *et al*. (2008), aceruloplasminemia, n= 1, moderate radiological or clinical change
	Haemers *et al*. (2004), aceruloplasminemia, n= 1, no radiological or clinical change	Kwiatkowski *et al*. (2012), idiopathic NBIA, n=1, T2* increase in SN/ dentate nuclei, Moderate clinical improvement	
	Loreal *et al*. (2002), aceruloplasminemia, n= 1, no radiological or clinical change	Forni *et al*. (2008), idiopathic NBIA, n=1, Reduced T2 hypointensities in BG, Moderate clinical improvement	
	Miyajima *et al*. (1997), aceruloplasminemia, n=1, T2* increase in striatum/ thalamus, moderate clinical improvement	Mariani *et al*. (2004), aceruloplasminemia, n= 1, no radiological or clinical change	
	Chinnery *et al*. (2007), neuroferritinopathy, n = 3, profound and refractory iron depletion. Clinical deterioration in one, no change in others		

* Disease Type, Number of Patients, Radiological and Clinlcal Outcome. Modified from [200] [201] and [202]
